# Prevalence of Enhanced Granular Expression of Thrombospondin Type-1 Domain-Containing 7A in the Glomeruli of Japanese Patients with Idiopathic Membranous Nephropathy

**DOI:** 10.1371/journal.pone.0138841

**Published:** 2015-09-22

**Authors:** Takamasa Iwakura, Naro Ohashi, Akihiko Kato, Satoshi Baba, Hideo Yasuda

**Affiliations:** 1 Internal Medicine I, Division of Nephrology, Hamamatsu University School of Medicine, Hamamatsu, Japan; 2 Blood Purification Unit, Hamamatsu University School of Medicine, Hamamatsu, Japan; 3 Division of Pathology, Hamamatsu University School of Medicine, Hamamatsu, Japan; Mario Negri Institute for Pharmacological Research and Azienda Ospedaliera Ospedali Riuniti di Bergamo, ITALY

## Abstract

Membranous nephropathy (MN) is a leading cause of nephrotic syndrome in adults. Autoantibodies against M-type phospholipase A2 receptor (PLA2R) and thrombospondin type-1 domain-containing 7A (THSD7A), which mainly belong to the IgG4 subclass, were reported as associated antibodies for the development of MN. Although PLA2R is a major target antigen for idiopathic MN, the prevalence of MN patients seropositive for PLA2R in Japan is lower than that in other countries. In this study, we conducted immunohistochemical analysis of the presence of THSD7A and PLA2R in renal specimens of MN patients to estimate the prevalence of THSD7A/PLA2R-related idiopathic MN in Japan. Enhanced granular expression of THSD7A and PLA2R was detected in 9.1% and 52.7%, respectively, of the patients with idiopathic MN. Although none of patients with secondary MN displayed enhanced granular expression of THSD7A, 5.4% of them had enhanced granular expression of PLA2R. In conclusion, the prevalence of enhanced granular expression of THSD7A in the glomeruli of Japanese patients with idiopathic MN was higher than the prevalence of MN patients seropositive for THSD7A in USA and Europe.

## Introduction

Membranous nephropathy (MN), which has idiopathic and secondary forms, is the main cause of nephrotic syndrome in adults [1]. Hepatitis B and C viruses, autoimmune diseases, thyroiditis, malignancies, and the use of certain drugs have been reported to cause secondary MN [2]. In contrast, the pathogenesis of idiopathic MN has been uncertain.

In 2009, Beck et al. first reported that M-type phospholipase A2 receptor (PLA2R) is a major target antigen for idiopathic MN and that approximately 70% of patients with idiopathic MN had autoantibodies to PLA2R in their serum [3]. After this discovery, there have been many reports on the prevalence of detection of anti-PLA2R antibodies in the serum of patients with idiopathic MN in many countries and regions, and this prevalence has been found to be in the range of 52% to 98% [4–12].

Western blotting, enzyme linked immunosorbent assay (ELISA), and indirect immunofluorescence (IIF) have been used to detect anti-PLA2R antibodies in serum [4–13]. Enhanced staining of the PLA2R antigen was found by immunohistochemistry of kidney biopsy specimens from patients who had these antibodies in their serum [8, 10, 12, 14]. In the study by Hoxha et al., all MN patients with anti-PLA2R antibodies in the serum showed enhanced expression of PLA2R in the glomeruli as determined by immunohistochemistry [8]. A meta-analysis of PLA2R-related MN has shown that the sensitivity and specificity of histological PLA2R staining in renal tissues for differentiating between idiopathic and secondary MN were similar to those of serological tests [15].

Akiyama and colleagues have recently reported that the prevalence of anti-PLA2R antibodies in Japanese patients with idiopathic MN was only 53% when determined by Western blotting [11]. This rate was lower than corresponding rates in a number of European and Asian countries as well as in USA, which has led researchers to the idea that another related factor may contribute to the development of MN in the rest of Japanese patients with idiopathic MN [11].

In 2014, Tomas et al. first described thrombospondin type-1 domain-containing 7A (THSD7A) as a new target antigen for idiopathic MN in Europe and USA [16]. In particular, in their study, anti-THSD7A antibodies were detected by Western blotting in the serum of 8–14% of idiopathic MN patients without anti-PLA2R antibodies (approximately in 2.5–5% of all patients with idiopathic MN assuming the prevalence of PLA2R-related MN to be 70%), and all the patients with anti-THSD7A antibodies in the serum showed enhanced granular expression of THSD7A in the glomeruli as had been previously observed for PLA2R.

Both anti-PLA2R and anti-THSD7A antibodies belong mostly to the IgG4 subclass [8, 16], and deposition of IgG4 on glomerular capillaries has been suggested as a potential marker for differentiating between primary and secondary MN [8, 17–20].

In the current study, we examined the prevalence of enhanced granular expression of THSD7A and PLA2R in the glomeruli of Japanese patients with idiopathic MN by immunohistochemistry. We also investigated positivity for IgG4 in idiopathic MN patients with or without enhanced granular expression of each of these antigens in the glomeruli, as well as in secondary MN patients. Our results suggest that the prevalence of THSD7A-related MN in Japanese patients with idiopathic MN is higher than that in Europe and USA [16], whereas that of PLA2R-related MN is similar to the previously reported values [11].

## Materials and Methods

### Patients

This study included 92 consecutive adult (age > 18 years) patients (48 men and 44 women) with the histologic diagnosis of MN established in our institution from 1995 to the present date. We examined paraffin-embedded tissue sections from patients with biopsy-proven MN and collected the clinical information and laboratory data at the time of biopsy by reviewing the patient’s medical records. All patients underwent rigorous screening for secondary causes of MN, which included serological analysis, physical examination, obtaining information on prescribed medications, and testing for malignancies. MN subjects with diseases associated with secondary MN were classified as having secondary MN. All other patients were classified as having idiopathic MN. Remission of proteinuria was defined as proteinuria <3.5 g/24 h and at least a 50% reduction from the time of inclusion. The study protocol was approved by the research ethics committee of Hamamatsu University School of Medicine (#E14-338), and the research was conducted in accordance with the ethical principles stated by the Declaration of Helsinki. The requirement for obtaining informed consent was waived by the research ethics committee based on the retrospective design of this study. Instead, a detailed disclosure of this study contents was published on the website of the research ethics committee. Patient records/information was anonymized and de-identified prior to analysis. This study was registered with the Japan Primary Registries Network (UMIN 000018079).

### Histological analysis

**Fig 1:**
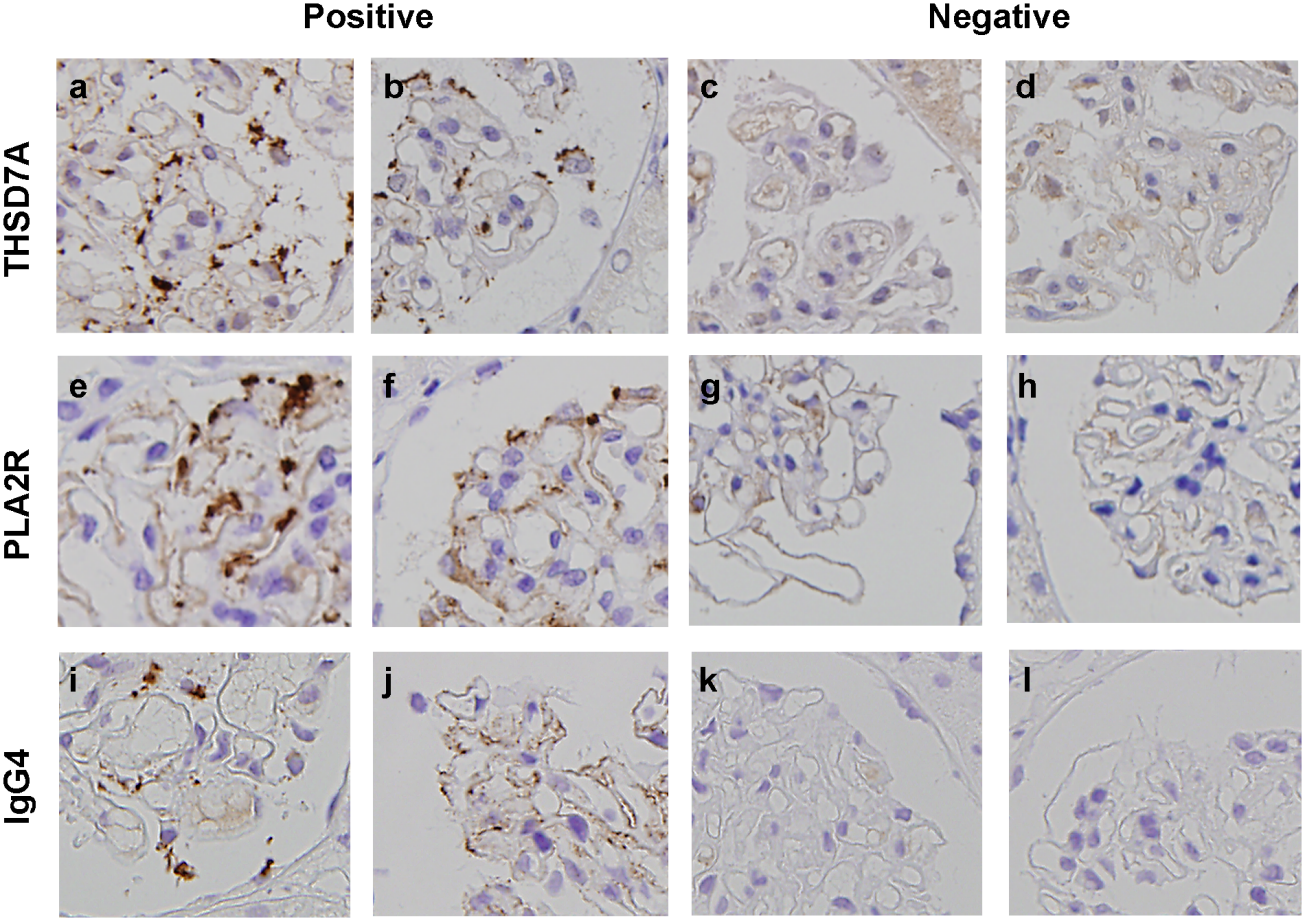
Examples of positive and negative staining for THSD7A, PLA2R, and IgG4. Positive staining for THSD7A (a, b), PLA2R (e, f), and IgG4 (i, j), and negative staining for THSD7A (c, d), PLA2R (g, h), and IgG4 (k, l) are shown.

For immunohistochemical analysis, the method described by Tomas et al. was used, with slight modifications [16]. Briefly, 2-μm paraffin-embedded sections of renal biopsy specimens from patients with MN were deparaffinised and rehydrated. Antigen retrieval was achieved by boiling in Histofine Antigen Retrieval Solution pH9® (Nichirei Bioscience, Tokyo, Japan) for 20 min at 98°C. Nonspecific binding was blocked with Protein Block Serum-Free® (#X0909; DAKO, Glostrup, Denmark) for 15 min at RT prior to incubation at 4°C overnight with rabbit anti-THSD7A (1:400, #HPA000923; Atlas Antibodies, Stockholm, Sweden) and anti-PLA2R (1:8000, #HPA012657; Atlas Antibodies) or mouse anti-IgG4 (1:500, #HP602, Binding Site, CA, USA) antibodies in phosphate buffered saline supplemented with 1% bovine serum albumin. The primary antibodies were reacted with Histofine Simple Stain MAX PO® (Nichirei Bioscience) and were visualised using a standard peroxidase-diaminobenzidine system. Nuclei were counterstained with haematoxylin. Primary antibodies were omitted for negative controls. Eight renal biopsies without MN were also evaluated by this staining procedure for minimal change disease (n = 1), focal segmental glomerulosclerosis (n = 1), IgA nephropathy (n = 1), membranoproliferative glomerulonephritis (n = 1), microscopic polyangiitis (n = 1), sarcoidosis (n = 1), obesity-related glomerulopathy (n = 1), and cholesterol emboli (n = 1). Positive staining was defined as the presence of dark brown granular deposits (i.e. enhanced staining) whose boundary was clear on the glomerular basement membrane. It was not difficult to distinguish between positive and negative staining in any case (Fig 1).

### Statistical analysis

All values were expressed as the mean ± s.d. Differences were analysed with either the chi-square statistic or the Mann-Whitney U test for comparison of 2 groups (Prism 6, GraphPad Software, San Diego, CA, USA). A P-value <0.05 was accepted as statistically significant.

## Results

### Characteristics of patients with secondary and idiopathic MN

The secondary MN group (n = 37) was composed of 15 patients with connective tissue disease, including systemic lupus erythematosus (n = 12) and overlap syndrome (n = 3); 3 patients with rheumatoid arthritis who were taking the anti-rheumatoid drug bucillamine; 8 patients with malignancies, including a lung, oesophageal, pancreatic, colon, prostate, and uterine neoplasm; 2 patients with strongly suspected multiple myeloma or lymphoma; 5 patients with hepatitis B virus (HBV) infection; 2 patients with hepatitis C virus (HCV) infection; 2 patients with thyroiditis; and 2 patients with anti-neutrophil cytoplasmic antibody-associated vasculitis. The 55 remaining patients were assigned to the idiopathic MN group. One patient in the idiopathic MN group and 9 patients in the secondary MN group were being treated with immunosuppressive agents at the time of biopsy.

### THSD7A and PLA2R immunohistochemistry in idiopathic MN and secondary MN patients

**Fig 2:**
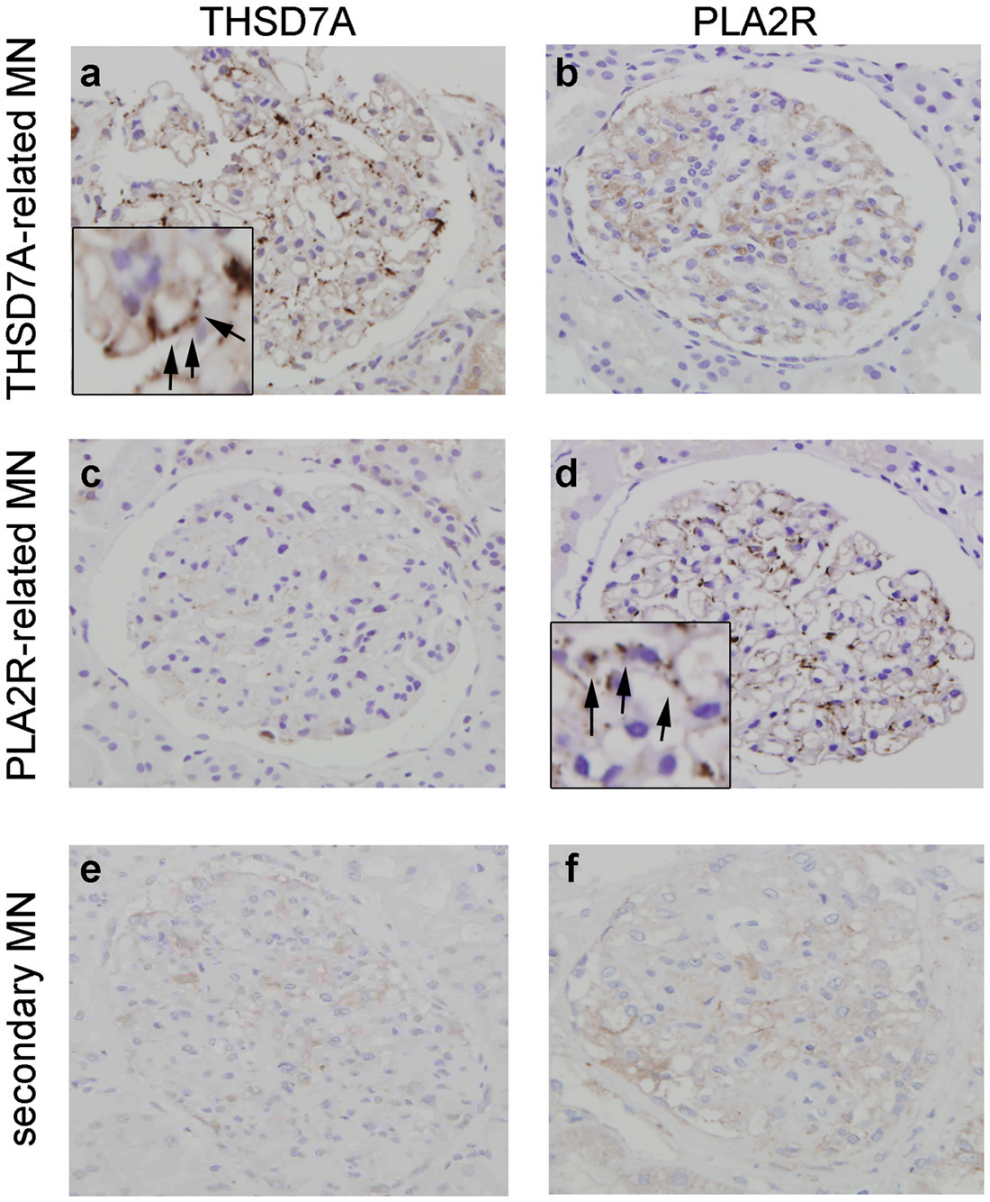
Immunohistochemical analysis for thrombospondin type-1 domain-containing 7A (THSD7A) and M-type phospholipase A2 receptor (PLA2R). Immunostaining for THSD7A (a, c, e) and PLA2R (b, d, f) in patients with idiopathic membranous nephropathy (a–d) and secondary membranous nephropathy (e, f). Original magnification: ×400. Inset, enhanced granular expression of THSD7A (a) and PLA2R (d) shown at a higher magnification (arrows). Abbreviations: THSD7A, Thrombospondin type-1 domain-containing 7A; PLA2R, M-type phospholipase A2 receptor; MN, membranous nephropathy.

Enhanced granular expression of THSD7A and PLA2R in the glomeruli was detected in 5 and 29 samples from the patients with idiopathic MN, respectively (Fig 2 and Table 1). Twenty-one samples from patients with idiopathic MN showed enhanced granular expression of neither THSD7A nor PLA2R. None of the samples from the patients with MN showed enhanced granular expression of both THSD7A and PLA2R. Only 2 samples from secondary MN patients who had HBV or malignancy showed enhanced granular expression of PLA2R in the glomeruli (data not shown). Granular expression of THSD7A was not enhanced in any other samples from the secondary MN patients. None of the samples from patients without MN showed enhanced granular expression of THSD7A or PLA2R (data not shown). The sensitivity and specificity of THSD7A/PLA2R positivity for detecting primary MN were 61.8% and 94.6%, respectively.

**Table 1:** Prevalence of enhanced granular expression of thrombospondin type-1 domain-containing 7A (THSD7A) and M-type phospholipase A2 receptor (PLA2R) in the glomeruli of patients with idiopathic membranous nephropathy (MN) and secondary MN.

	THSD7A+ Number (%)	PLA2R+ Number (%)	both negative Number (%)
Idiopathic MN	5 (9.1)	29 (52.7)	21 (38.2)
Secondary MN	0 (0)	2 (5.4)	35 (94.6)

### Characteristics of patients with enhanced granular expression of THSD7A or PLA2R in the glomeruli

A comparison of the clinical characteristics at the time of biopsy in patients with enhanced granular expression of each antigen in the glomeruli (Table 2) did not reveal any significant difference between the two groups with respect to sex and urinary protein, serum albumin, and serum creatinine levels. The patients with enhanced granular expression of THSD7A were younger (P = 0.02). In patients with enhanced granular expression of THSD7A and PLA2R, 4 of 5 (80.0%) and 21 of 29 (72.4%) patients, respectively, were treated with immunosuppressive agents. Among patients treated with immunosuppressive agents, 1 with enhanced granular expression of THSD7A and 5 with enhanced granular expression of PLA2R were transferred to other hospitals; therefore, follow-up data could not be collected from these patients. In the remaining patients, remission of proteinuria 1 year after the initiation of immunosuppressive agents was achieved in 2 of 3 (66.7%) and 13 of 16 (81.3%) patients with enhanced granular expression of THSD7A and PLA2R, respectively.

**Table 2:** Characteristics of idiopathic membranous nephropathy (MN) patients with enhanced granular expression of thrombospondin type-1 domain-containing 7A (THSD7A) or M-type phospholipase A2 receptor (PLA2R) in the glomeruli.

	THSD7A+	PLA2R+	P value
Age in years	42.4 ± 19.9	62.0 ± 11.2	0.02
Gender male/female (% male)	2/3 (40.0%)	19/10 (65.5%)	0.81
Proteinuria (g/day)	4.8 ± 2.7	2.5 ± 0.6	0.3
Serum albumin (g/dl)	1.9 ± 1.0	1.7 ± 2.2	0.09
Serum creatinine (mg/dl)	0.7 ± 0.2	0.9 ± 0.3	0.3

The characteristics of the secondary MN patients with enhanced granular expression of PLA2R in the glomeruli were as follows. The patient with HBV and enhanced granular expression of PLA2R entered a spontaneous remission without antiviral therapy. Although the MN patient with malignancy (i.e., colon cancer) and enhanced granular expression of PLA2R was not cured of the tumour because of liver and lymph node metastasis, the patient showed complete spontaneous remission without therapy.

Disease severity was assessed by evaluating proteinuria and serum albumin and serum creatinine levels at the time of biopsy. The chi-square statistic was used to investigate the proportion of sex, and the Mann-Whitney U test was used for the other variables. Data are expressed as means ± s.d.

### IgG4 immunohistochemistry

Immunohistochemistry showed that 3 of the 5 and 28 of the 29 renal biopsy specimens from the idiopathic MN patients with enhanced granular expression of THSD7A and PLA2R, respectively, were positive for IgG4 (Table 3). Four renal biopsy samples from the idiopathic MN patients without enhanced granular expression of THSD7A or PLA2R were positive for IgG4. In the secondary MN group, 2 patients with enhanced granular expression of PLA2R had enhanced granular expression of IgG4, whereas the remaining patients did not show such enhanced expression. The sensitivity and specificity of IgG4 positivity for detecting primary MN were 63.6% and 94.6%, respectively.

**Table 3:** Numbers of patients with enhanced granular expression of IgG4 among thrombospondin type-1 domain-containing 7A (THSD7A)-positive, M-type phospholipase A2 receptor (PLA2R)-positive, and THSD7A/PLA2R-negative patients with idiopathic MN and secondary membranous nephropathy (MN).

		THSD7A+ Number	PLA2R+ Number	both negative Number
Idiopathic MN	IgG4-positive	3	28	4
	IgG4-negative	2	1	17
Secondary MN	IgG4-positive	0	2	0
	IgG4-negative	0	0	35

## Discussion

The present study revealed that the prevalence of enhanced granular expression of THSD7A in the glomeruli detected by immunohistochemical analysis of biopsy samples was 9.1% in the studied cohort of Japanese patients with idiopathic MN. Additionally, the prevalence of enhanced granular expression of THSD7A was 19.2% in the idiopathic MN patients without enhanced granular expression of PLA2R. These values are higher than those reported by Tomas et al. [16]. There are several possible reasons for this discrepancy. First, differences in genetic background and environmental conditions may be involved. Thus, the above study was performed in Europe and USA, and Asian patients were likely a minority in this cohort. Second, there could be a contribution from methodological variations. Tomas et al. used both Western blotting and immunohistochemistry to show that only MN patients with anti-THSD7A antibodies in the serum had enhanced granular expression of THSD7A in the glomeruli. However, specimens from only 5 patients were available in their study. Therefore, it is not clear whether all the patients with anti-THSD7A antibodies in the serum also have enhanced granular expression of THSD7A in the glomeruli, and, conversely, the samples with enhanced granular expression of THSD7A in the glomeruli may not necessarily have anti-THSD7A antibodies in the serum. However, the presence of cases with anti-THSD7A antibodies only in the serum in our study would increase the prevalence of THSD7A-related MN. Third, the timing of evaluation could be associated with the differences in prevalence. We assessed the prevalence at the time of the biopsy, whereas in the study of Tomas et al., the serum was collected from MN patients between 0 and 87 months from the time of biopsy. Furthermore, Tomas and colleagues also reported that the percentage of anti-THSD7A antibody-positive serum samples was higher if these samples were obtained close to the time of the biopsy as compared to the serum samples procured long after [16]. It is possible that the serum titres of the anti-THSD7A antibodies decreased in some of these patients after the biopsy because they were treated with immunosuppressive agents, resulting in the lower prevalence of THSD7A-related MN in the previous study.

The prevalence of enhanced granular expression of PLA2R in the glomeruli in our study was 52.7%, which is similar to the value reported by Akiyama et al. [11], indicating that both Western blotting and immunohistochemistry are adequate methods to diagnose patients with PLA2R-related MN. Previous reports from other countries, excluding a German study that showed that 52% of idiopathic MN patients had anti-PLA2R antibodies as detected by IIF [6], demonstrated that the prevalence of anti-PLA2R antibodies in the serum was 69–98%. Although the proportion of patients with anti-PLA2R antibodies in the German study was similar to that in the Japanese study, a large number of the patients in the German study were treated with immunosuppressive agents at the time of serum collection, whereas in the previous Japanese study, the serum samples were collected at the time of biopsy, with no patients being treated with immunosuppressive agents. In the present study, only 1 patient with idiopathic MN was receiving immunosuppressive agents at the time of biopsy. The authors of the German report subsequently performed a prospective study that revealed that 84% of the renal tissues from idiopathic MN patients who did not receive any immunosuppressive agents had enhanced expression of PLA2R as determined by immunohistochemistry, and anti-PLA2R antibodies in the serum were detected by IIF in 98.4% of those patients [8]. This result is likely related to the fact that immunosuppressive agents reduce the level of anti-PLA2R antibodies [21]. Therefore, the prevalence of Japanese patients with idiopathic MN positive for anti-PLA2R antibodies is lower compared to other countries [3–5, 7–10, 12, 13].

None of the samples of the secondary MN patients showed enhanced granular expression of THSD7A, whereas 1 of the 5 samples from the patients with HBV and 1 of the 8 samples from the patients with malignancies showed enhanced granular expression of PLA2R. In agreement with the report by Qin et al. [5], the MN patients with HBV or malignancy and enhanced granular expression of PLA2R entered remission without antiviral therapy or tumour treatment. In addition, the renal tissues from these 2 patients showed enhanced granular expression of IgG4 in the glomeruli. IgG4 positivity is thought to be indicative of idiopathic rather than secondary MN [17–20], suggesting that these 2 patients are likely to have coincidental occurrence of idiopathic MN and diseases associated with secondary MN.

Differentiating between primary and secondary MN is very important. In this study, the sensitivity of THSD7A/PLA2R for detecting primary MN was 61.8%, and therefore the remaining 38.2% of the patients likely had idiopathic MN of unknown aetiology. In these patients, the levels of enhanced granular expression of THSD7A/PLA2R may be below the detection limit of immunohistochemistry, they might have other related autoantibodies, or their disease may be due to undetected secondary causes. The sensitivity of IgG4 staining for detecting primary MN was 63.6%, indicating that immunohistochemistry for THSD7A/PLA2R is similar to immunohistochemistry for IgG4 in terms of detecting primary MN. However, it is worth noting that the glomeruli of some patients were IgG4-positive but PLA2R/THSD7A-negative, whereas in other cases, they were IgG4-negative but THSD7A- or PLA2R-positive. There are several possible explanations for these phenomena. First, although it has been reported that the level of anti-PLA2R antibodies correlates with the level of IgG4, patients with high levels of anti-PLA2R antibodies and low levels of IgG4 have been described [7, 13], and our subject with enhanced granular expression of THSD7A or PLA2R but without enhanced expression of IgG4 could belong to this pool. Second, an IgG subclass switch in the course of the disease progression has been suggested for idiopathic MN [22]. Huang et al. reported that IgG1 was the dominant subclass in the early stage of idiopathic MN, whereas IgG4 dominated in the late stage. Therefore, patients with enhanced granular expression of THSD7A or PLA2R and without enhanced granular expression of IgG4 might be diagnosed at the early stage. Third, other related autoantibodies may be present in patients with enhanced granular expression of IgG4 but without enhanced granular expression of THSD7A/PLA2R.

This study has several limitations. First, this single-centre study included only one geographical region, and its sample size was relatively small compared to the previous study. Second, THSD7A and PLA2R-related MN were examined by immunohistochemistry only. Therefore, patients with only anti-THSD7A or anti-PLA2R antibodies in the serum might have remained unidentified. Future studies using immunohistochemistry for THSD7A and PLA2R detection in other regions of Japan are needed. Moreover, it is essential to conduct further validation studies examining the rate of concordance in detecting PLA2R/THSD7A-related MN between the findings obtained by immunohistochemistry of renal tissues and by serological tests such as Western blotting, ELISA, and IIF. Third, we did not perform methods such as isotype-matching and pre-absorption of primary antibody with the human THSD7A antigen. This may influence our results. Fourth, our assignment of secondary MN by existence of diseases associated with secondary MN was not perfect because 2 patients with an enhanced expression of PLA2R and IgG4 were included in the secondary MN group. However, the prevalence of PLA2R- and THSD7A-related MN did not greatly differ even if these 2 patients were included in the idiopathic MN group.

## Conclusions

We determined that the prevalence of enhanced granular expression of THSD7A in the glomeruli of Japanese patients with idiopathic MN is 9.1%, which is higher than the corresponding value in the previous study conducted in Europe and USA. In addition, the prevalence of enhanced granular expression of PLA2R in the glomeruli of these patients was in agreement with the data from the previous Japanese study. The combined prevalence of patients with enhanced granular expression of THSD7A or PLA2R (61.8%) was lower than the estimated combined prevalence of MN patients with the serum positive for anti-PLA2R or anti-THSD7A antibodies in other countries.
